# Combined BSA-seq and RNA-seq approaches reveal candidate genes associated with seed weight in *Brassica napus*

**DOI:** 10.3389/fpls.2025.1678464

**Published:** 2025-09-16

**Authors:** Xinxin Geng, Fengling Yang, Wenhua Tang, Ying Wang, Shan Fu, Zichen Yu, Wanli Cheng, Liang Chen, Xiaomeng Xue

**Affiliations:** ^1^ Hubei Key Laboratory of Edible Wild Plants Conservation & Utilization, Hubei Normal University, Huangshi, China; ^2^ College of Life Sciences, Hubei Normal University, Huangshi, China; ^3^ Hubei Engineering Research Center of Special Wild Vegetables Breeding and Comprehensive Utilization Technology, Hubei Normal University, Huangshi, China

**Keywords:** seed weight, *Brassica napus*, BSA-seq, RNA-seq, candidate genes

## Abstract

*Brassica napus*, a globally significant oilseed crop of the *Brassicaceae* family, serves as a major source of vegetable oil and biofuel. Seed size/weight is a crucial agronomic trait that directly determines crop yield. However, the genetic mechanisms underlying seed weight in *B*. *napus* has not been fully understood. In this study, R140 with extremely low thousand-seed weight (2.6g) was crossed with Zhongshuang 11 (4.9g) to construct an F_2_ population. Five major genomic regions on chromosomes A06 (43.88-44.63Mb), A08 (27.63-27.68Mb), A09 (55.32-55.46Mb, 57.33-57.58Mb), and C07 (29.40-29.60Mb) were identified as candidate loci of seed weight via BSA-seq approach. A total of 204 genes were annotated within the candidate regions, including 103 non-synonymous mutant genes and 26 frameshift mutant genes identified between parental lines. Among them, 21 DEGs were screened through RNA-seq analysis of the developing seeds in both parents. However, only 8 genes exhibited mutations in their coding or upstream sequences, which were characterized as the candidate genes associated with the small seed phenotype of R140. An auxin response factor18 coding gene (*BnARF18*) exhibited significantly differential expression between parents. Analysis of the promoter element variations revealed that the MYC-motif, implicated in gene expression regulation, and the WUN-motif, associated with cell differentiation and proliferation control, likely serve as key regulatory motifs responsible for the differential expression levels of *BnARF18* between the two parental lines. It was therefore considered to be the most likely candidate gene. In conclusion, this study provides clues for elucidating the molecular mechanism of seed weight regulation in *Brassica napus*.

## 1 Introduction

*Brassica napus*, a significant oil crop belonging to the *Brassicaceae* family, represents one of the three primary cultivation types of rapeseeds (Ford et al., 2012). As the second largest source of vegetable oil in the world, it has strategic importance in ensuring food and oil security, promoting sustainable agriculture, and advancing bioenergy development (Rana et al., 2004; Lu et al., 2011). As a heterozygous tetraploid plant, its genome research also provides an important model for plant genetics. Therefore, elucidating the genetic mechanisms underlying important agronomic traits, such as seed weight, is essential for developing high-yield varieties that can meet the increasing global demand for vegetable oil while addressing challenges of sustainable agriculture.

Seed weight is one of the three major factors that contribute to the yield per plant (effective number of siliques per plant, number of seeds per silique, and seed weight), and an important breeding objective in *Brassica napus*. The determination of seed weight primarily relies on two essential morphological characteristics: seed size and plumpness. Notably, the size of seeds is influenced by the synergistic development of three critical components: embryo, endosperm, and seed coat (Zhang et al., 2018). In addition, large seeds could provide more nutrients during seedling germination, enhance adaptability to the environment (Ding et al., 2012), own a high germination rate (Geritz et al., 1999; Adamski et al., 2009), be resistant to mechanized harvesting (Qing et al., 2021) and increase oil content (Zhang et al., 2022).

Seed weight, a classic quantitative trait governed by multiple genes with large additive and non-additive effects (Grant and Beversdorf, 1985; Ishaq et al., 2017; Karikari et al., 2019; Contreras et al., 2008), has been extensively studied. Numerous QTLs and genes influencing seed weight have been characterized in a wide range of crops, notably rice (Li et al., 2024a; Wang et al., 2023; Kumar et al., 2020), soybean (Xu et al., 2023; Goettel et al., 2022; Gao et al., 2024), peanut (Yang et al., 2023; Li et al., 2021; Fang et al., 2023), maize (Liu et al., 2020; Wang et al., 2020a), wheat (Wang et al., 2022; Zeng et al., 2023) and etc. In *Brassica napus*, a set of 119 QTLs distributed on all 19 chromosomes were identified for seed weight (Dong et al., 2023; Meng et al., 2025). Yang et al. (2012) discovered 9 QTLs for seed weight on Chromosomes A01, A06, A07, A09 and C09 with 1.3%-28.2% PVE (phenotypic variation explained). Dong et al. (2018) detected 20 significant seed weight-associated SNPs on chromosomes A01, A04, A09, C02, and C06 by GWAS (genome-wide association analysis). A major QTL mapping to chromosome A09 (25.40-25.98Mb) was identified for seed weight by BSA-seq analysis (Geng et al., 2016). Recently, 15 QTLs were detected for seed weight, among which the major QTL *qSW-A03* was delineated to a 59kb genomic region through fine-mapping (Meng et al., 2025). Although many QTLs for seed weight have been reported, only two genes, *BnaA9.CYP78A9* and *BnaA9.ARF18*, have been cloned through QTL mapping approaches.

Numerous genes have been identified to influence seed weight through coordinated regulation of cell proliferation and expansion-related signaling pathways, including Brassinoid metabolic pathway, auxin signaling pathways, ubiquitin proteasome metabolic pathways, G-protein signaling pathways, (HAIKU) IKU pathway, MAPK signaling pathway and transcriptional regulatory factors (Zhang et al., 2023; Li et al., 2019). In *Brassica napus*, auxin responsive factor *BnARF18* negatively regulated seed weight by affecting the growth of silique in the auxin signaling pathways (Liu et al., 2015). *BnDA1* negatively regulated oilseed weight by regulating the proliferation of seed coat cells in the ubiquitin proteasome metabolic pathways (Wang et al., 2017). *SHB1* and *HAIKU2* increased final seed weight by regulating endosperm proliferation in the IKU pathway (Xiao et al., 2016). However, most of the reported genes were obtained by homologous cloning. Therefore, comprehending the genetic mechanism and discovering new loci/gene governing the seed weight in rapeseed is crucial for developing new high-quality, high-yield rapeseed varieties.

Bulked Segregant Analysis combined with next-generation sequencing (BSA-seq) is an efficient genetic mapping method used to identify genomic regions associated with target traits. Individuals with extreme phenotypes from a segregating population are selected to form two contrasting DNA pools. These pools are deeply sequenced, and genomic regions linked to the trait are detected by analyzing significant differences in allele frequencies, such as SNPs and Indels, between the pools. BSA-seq streamlines gene localization, enabling rapid focus on candidate regions (Ye et al., 2022a, b). Transcriptome refers to the collection of all gene transcription products in a specific cell, tissue, or organism at a certain developmental stage or physiological condition. Through transcriptome sequencing technology, the sequence and expression level of these RNAs can be measured at high throughput, thus comprehensively analyzing the expression of genes and discovering genes that play key roles and differentially expressed genes in specific states (Wang et al., 2019; Wu et al., 2021). The combined analysis of BSA-seq and RNA-seq has significant value in rapidly identifying regulatory genes for known traits and analyzing the genetic mechanisms of complex traits (Wang et al., 2023, 2024a; Yu et al., 2024; Lei et al., 2024). Ye et al. (2022a) screened a total of 59 DEGs in the candidate regions, and 4 DEGs were identified as the most likely candidates responsible for the albino phenotype through BSA-seq and RNA-seq in *Brassica napus*. Yang et al. (2024) revealed through comprehensive analysis of BSA-seq and RNA-seq data that 9 genes on chromosome A10 and one gene on chromosome A05 are potential candidate genes for controlling mustard seed weight in *Brassica juncea*. Ye et al. (2022b) obtained seven candidate DEGs associated with plant Architecture in *Brassica napus* by combined BSA-seq based mapping and RNA-seq profiling. Wang et al. (2024b) identification of five candidate yellow seed color genes using BSA-seq and RNA-seq in *Brassica juncea* L.

In this study, a small seed natural mutant R140 was discovered and hybridized with a large seed weight variety Zhongshuang 11 (ZS11) to obtain a hybrid offspring population. BSA-seq was performed on the small seed pool, large seed pool and their parents in the F_2_ population to determine the genomic intervals associated with seed weight. By combining RNA-seq, seed weight related genes were further screened, providing a theoretical basis for improving the seed weight regulation network of *Brassica napus* and enhancing the breeding process of ornamental rapeseed.

## 2 Materials and methods

### 2.1 Plant materials and growth conditions

In this study, ZS11 (♀) and a mutant line R140 (♂) were used as the parental materials, where ZS11 exhibited a normal thousand-seed weight (TSW) phenotype. R140 is a spontaneous mutant, exhibiting a low TSW phenotype. ZS11 (P1) and R140 (P2) were crossed to produce F_1_ progeny, and then the F_1_ progeny was used to generate an F_2_ segregating population by self-crossing. The F_1_, F_2_ generations were planted in the experimental field at Hubei Normal University with row spacing of 40 cm and plant spacing of 25 cm under standard conditions during 2023-2024, Huangshi, China. While the two parental lines were planted at the same field during 2022-2024. To avoid the effects of different pollination methods on seed weight, all plants for phenotyping were self-pollinated by bagging.

### 2.2 Trait evaluation

The weight of 1000 seeds were measured as thousand-seed weight (TSW) for each sample. Prior to measurement, all seed samples were air-dried at room temperature until a constant weight was achieved to ensure standardized moisture content across all samples. The fully dried seeds of two parents, F_1_ and F_2_ individuals were used to measure seed weight trait after harvesting. In order to further understand the impact of five other yield related traits, including plant height, number of siliques per plant, silique length, number of seeds per silique, and seed yield per plant, five representative plants of ZS11 and R140 lines were used for trait evaluation as mentioned earlier (Ding et al., 2012).

### 2.3 Paraffin section analysis

Mature seeds of R140 and ZS11 were quickly placed in FAA fixative for fixation. Different gradients of ethanol were selected for dehydration treatment for 30 minutes each time. Different ratios of xylene, ethanol, and xylene were used for transparency treatment. Then, different ratios of xylene, paraffin wax mixture, and paraffin pure liquid were selected for wax immersion treatment. Finally, the seeds were embedded in the wax solution and cooled. Subsequently, slicing, unfolding, baking, and dewaxing were carried out, and staining was performed using safranin and solid green dye solutions. Finally, the films were dehydrated, cleared, sealed, and labeled after microscopic examination.

### 2.4 Bulked segregant analysis sequencing

According to the distribution of seed weight in the F_2_ population, 30 small-seed plants and 30 large-seed plants were collected. The CTAB method was used to extract genomic DNA from the parents ZS11, R140, and 60 selected rapeseed leaf samples. The DNA concentration and quality were estimated using a NanoDrop 2000 spectrophotometer (Thermo Scientific, Massachusetts, USA) and 1.5% agarose gel electrophoresis. DNA from 30 large-seed F_2_ plants was pooled to create a large-seed library (large-seed pool), while DNA from 30 small-seed F_2_ plants formed a small-seed library (small-seed pool). The prepared two pools, and two parents were sent to Biomarker Technologies Co. (Beijing, China) for library construction. Sequencing was performed on the Illumina HiSeq X Ten platform (Illumina, California, USA), and the raw data was firstly filtered through Fastp software to remove low-quality readings and adapter sequences. Raw sequences were transformed into clean reads after data processing and the clean readings were mapped to the reference genome http://cbi.hzau.edu.cn/bnapus/. Single nucleotide polymorphisms (SNPs) and insertion/deletion mutations (Indels) were analyzed using the HaplotypeCaller module in Genome Analysis Kit (GATK) software (v3.8). The average distribution of SNPs on 19 chromosomes of *Brassica napus* was calculated using sliding window analysis method, with a window size of 1Mb and an increment of 100kb.

Calculating SNP-index is an association analysis method to find the significant differences of genotype frequency between the pools, indicated by Δ(SNP/Indel-index). In the preset project, P and M stand for male and female parents, while aa and ab mean small-seed pool and large-seed pool, respectively. The Δ(SNP/Indel-index) was calculated as follows: SNP/Indel-index(aa)=Maa/(Paa+Maa), SNP/Indel-index(ab)=Mab/(Pab+Mab),Δ(SNP/Indel-index)=SNP/Indel-index(aa)-SNP/Indel-index(ab), in which Maa was the depth of aa population derived from Maa and Paa was the depth of aa population derived from P; Mab indicates the depth of ab population derived from Mab and Pab indicates the depth of ab population derived from P. The method of local weighted regression was adopted to obtain the correlation threshold, and only the intervals whose fitted the Δ(SNP-index) and Δ(Indel-index) values both exceeded their corresponding thresholds were defined as trait related candidate regions.

Gene function of candidate association region was annotated based on the following databases with BLAST software: Nr (NCBI non-redundant protein sequences); Pfam (Protein family); KOG/COG (Clusters of Orthologous Groups of proteins); Swiss-Prot (A manually annotated and reviewed protein sequence database); KO (KEGG Ortholog database) and GO (Gene Ontology).

### 2.5 RNA sequencing analysis

Total RNA was extracted from seeds of ZS11 and R140 strains at 7, 14, 21, 28, and 35 days after flowering using Trizol assay kit, respectively. The concentration and purity of these RNA samples were measured using a NanoDrop 2000 spectrophotometer (Thermo Scientific, Massachusetts, USA) and 1.5% agarose gel electrophoresis. For each parent line, equal amounts of RNA from these five developmental stages were pooled to form a single composite sample for library construction and subsequent RNA-seq analysis. Each sample has 3 biological replicates. A cDNA sample preparation kit was used to construct 6 cDNA libraries, and the concentration and mass of each cDNA library were measured using the Illumina HiSeq2000 platform, Agilent 2100 Bioanayzer, and ABI Step one Plus Real Time PCR System, respectively. The raw RNA-seq data was processed to remove low-quality data, adapter data, and high content of unknown bases. The high-quality and clean data of each sample is aligned to the reference genome sequence http://cbi.hzau.edu.cn/bnapus/, and only uniquely aligned reads are considered for gene expression analysis. Use RESM program to calculate differential gene expression and transcription abundance (expressed as FPKM values). Genes with FPKM<1 in all samples were excluded from subsequent analysis. Differentially expressed genes (DEGs) were identified using DESeq2 based on two criteria: false discovery rate (FDR)<0.01 and | log2fold change (FC) |>1.

Gene Ontology (GO) and Kyoto Encyclopedia of Genes and Genomes (KEGG) functional annotations for DEGs were retrieved using blast2go3 and blastx/blastp searches against the GO database4 and KEGG database,5 respectively. GO terms with *p* values ≤ 0.0001 and KEGG pathways with Q-values ≤ 0.05 were considered to be significantly significant.

### 2.6 Quantitative real-time PCR analysis

Total RNA extracted for RNA-seq was used for conducting qRT-PCR. Seven genes were randomly selected to validate RNA sequencing data by qRT-PCR. The primer pairs were designed using Primer 5.0 (Thermo Fisher, MA, United States). Details of the primers used are listed in **Supplementary Table 1**. The EasyScript^®^ All-in-One First-Strand cDNA Synthesis SuperMix for qPCR (One-Step gDNA Removal) kit (TIANGEN, China) was used for reverse transcription. qRT-PCR was performed on an ABI StepOne™ Real-time PCR System (Applied Biosystems, CA, United States). All qRT-PCR experiments included three technical replicates and three biological replicates. The *Bnactin* gene was used as an internal control.

## 3 Results

### 3.1 Phenotypic analysis of seed weight in parents, F_1_ and F_2_ population

**Figure 1:**
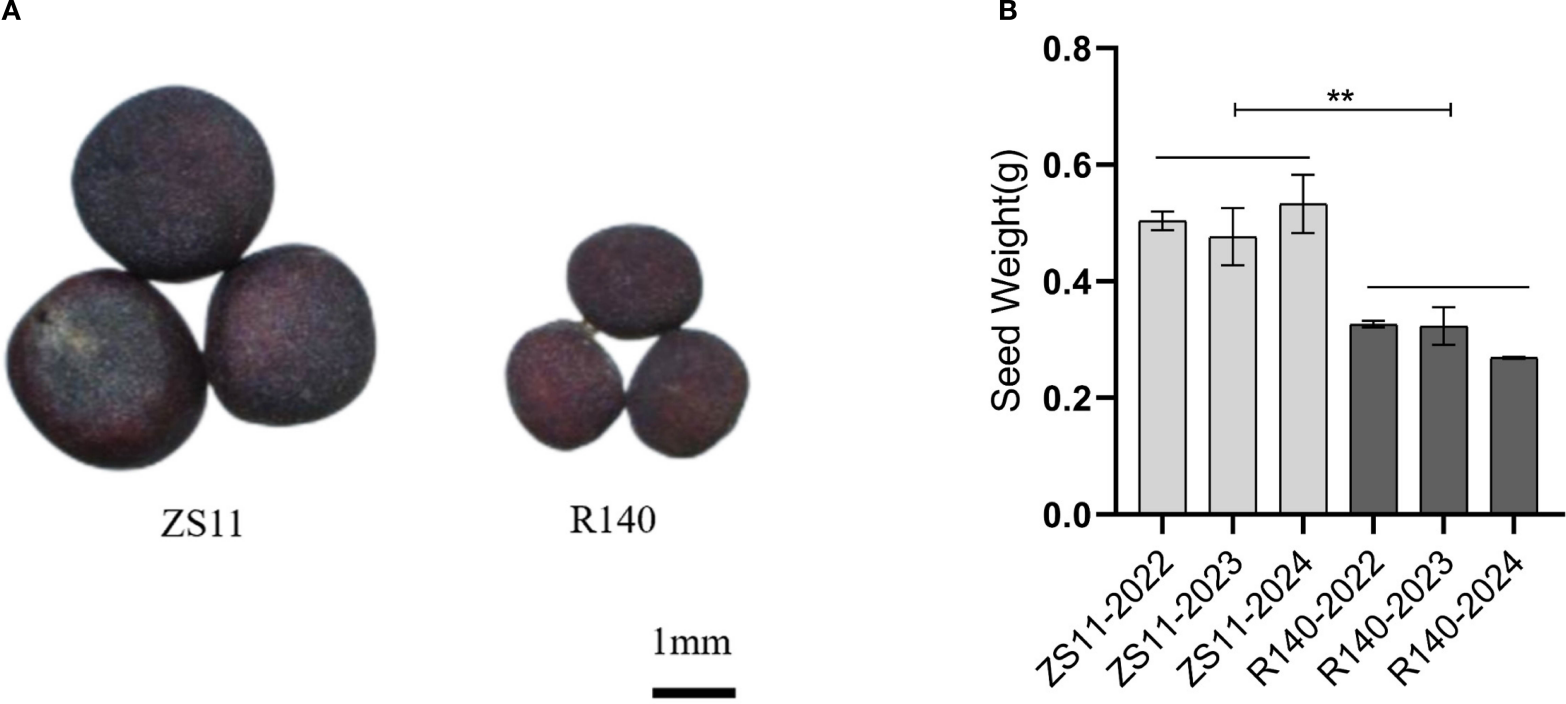
Phenotypic characterization of *B*. *napus* lines Zhongshuang 11 (ZS11) and R140. **(A)** Comparisons in seed size in matured seeds of ZS11 and R140. **(B)** Comparison in TSW of matured seeds between ZS11 and R140. The data are the means ± SE (n = 10). *p*-value denotes a significant difference between two lines in TSW.

**Figure 2:**
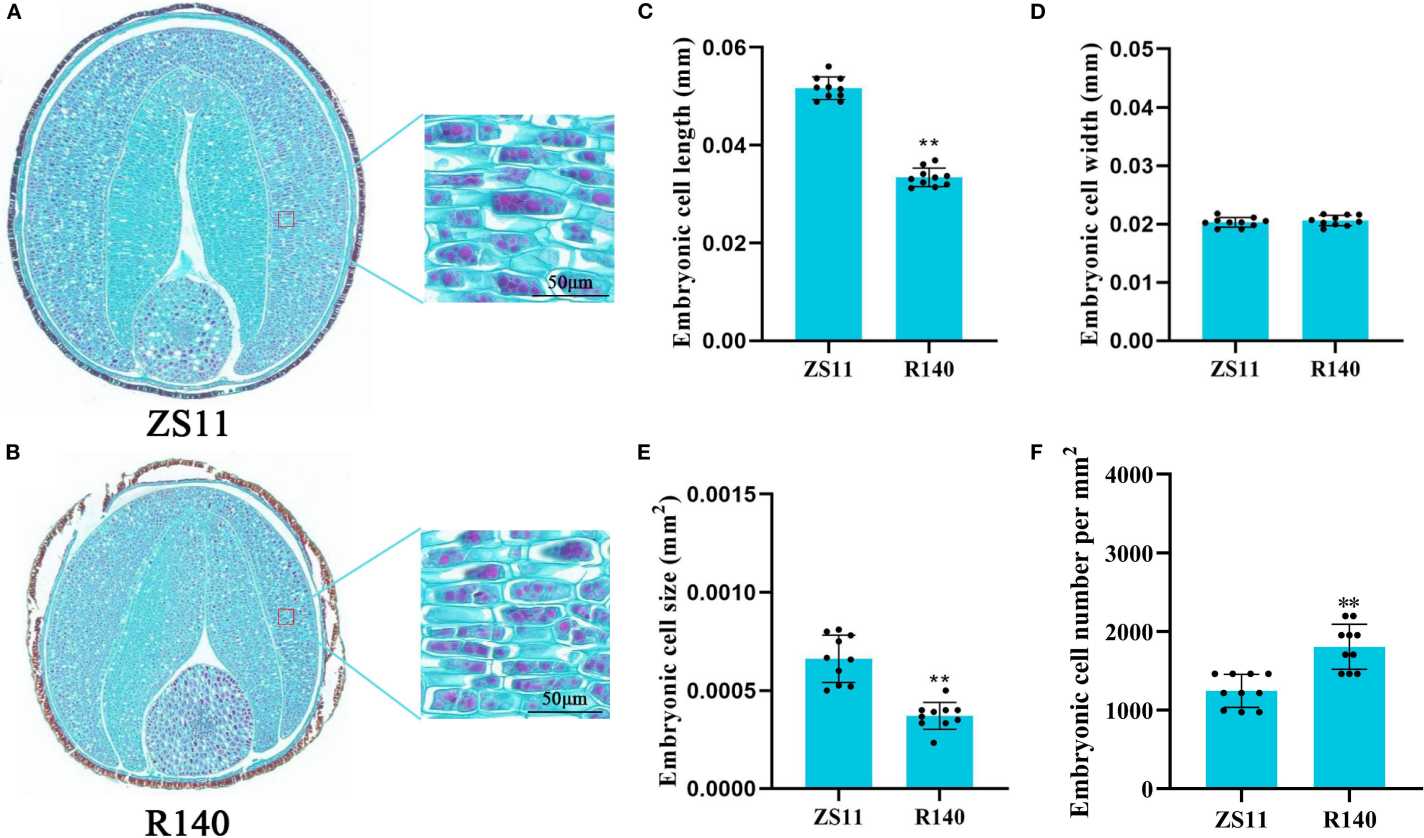
Comparison of cotyledon cell size in matured seeds of Zhongshuang 11(ZS11) and R140 through paraffin sections. **(A)** The phenotype of cotyledon cells in matured seeds of ZS11. Scale bar = 50 μm. **(B)** The phenotype of cotyledon cells in matured seeds of R140. Scale bar = 50 μm. **(C)** Cotyledon cell length in matured seeds of ZS11 and R140. Error bars represent the standard deviation (SD) of ten biological replicates. Asterisks indicate statistically significant differences (** means *p* < 0.01; Student’s t-test). **(D)** Cotyledon cell width in matured seeds of ZS11 and R140. Error bars represent the standard deviation (SD) of ten biological replicates. Asterisks indicate statistically significant differences (** means *p* < 0.01; Student’s t-test). **(E)** Cotyledon cell size in matured seeds of ZS11 and R140. Error bars represent the standard deviation (SD) of ten biological replicates. Asterisks indicate statistically significant differences (** means *p* < 0.01; Student’s t-test). **(F)** Cotyledon cell number in matured seeds of ZS11 and R140. Error bars represent the standard deviation (SD) of ten biological replicates. Asterisks indicate statistically significant differences (** means *p* < 0.01; Student’s t-test).

The average TSW of R140 is 2.6g, which is significantly lower than that of ZS11(4.9g) (**Figures 1A, B**). In general, seed size and weight are determined by cell division or cell expansion in plants (Garcia et al., 2005). To investigate the cause of the decreased seed weight observed in R140, we compared the cell numbers and cell area in seed of R140 and ZS11 plants. Upon observing the cross-sections of the seeds, we found that the difference in the size of R140 and ZS11 seeds originated from the cotyledons. After further observing and statistically analyzing the cotyledon cells of two parents, it was showed that the cell area of R140 was significantly smaller than that of ZS11 (**Figures 2A-F**). It demonstrated the reduction in seed size/seed weight of mutant R140 was due to the small cell of cotyledons at the cellular level.

Simultaneously, we also investigated several yield related traits of two parents, ZS11 and R140. Compared with ZS11, R140 showed a significant reduction in plant height and seed yield per plant (**Supplementary Figures 1A, E**) and a very significant reduction in silique length (**Supplementary Figure 1C**). However, there was no significant difference in silique number per plant and seed number per silique (**Supplementary Figures 1B, D**), indicating that the mutant R140 showed a decrease in seed weight accompanied by a decrease in silique length and plant height. The decrease in seed yield per plant was mainly caused by seed weight, rather than the other two factors, silique number per plant and seed number per silique.

**Figure 3:**
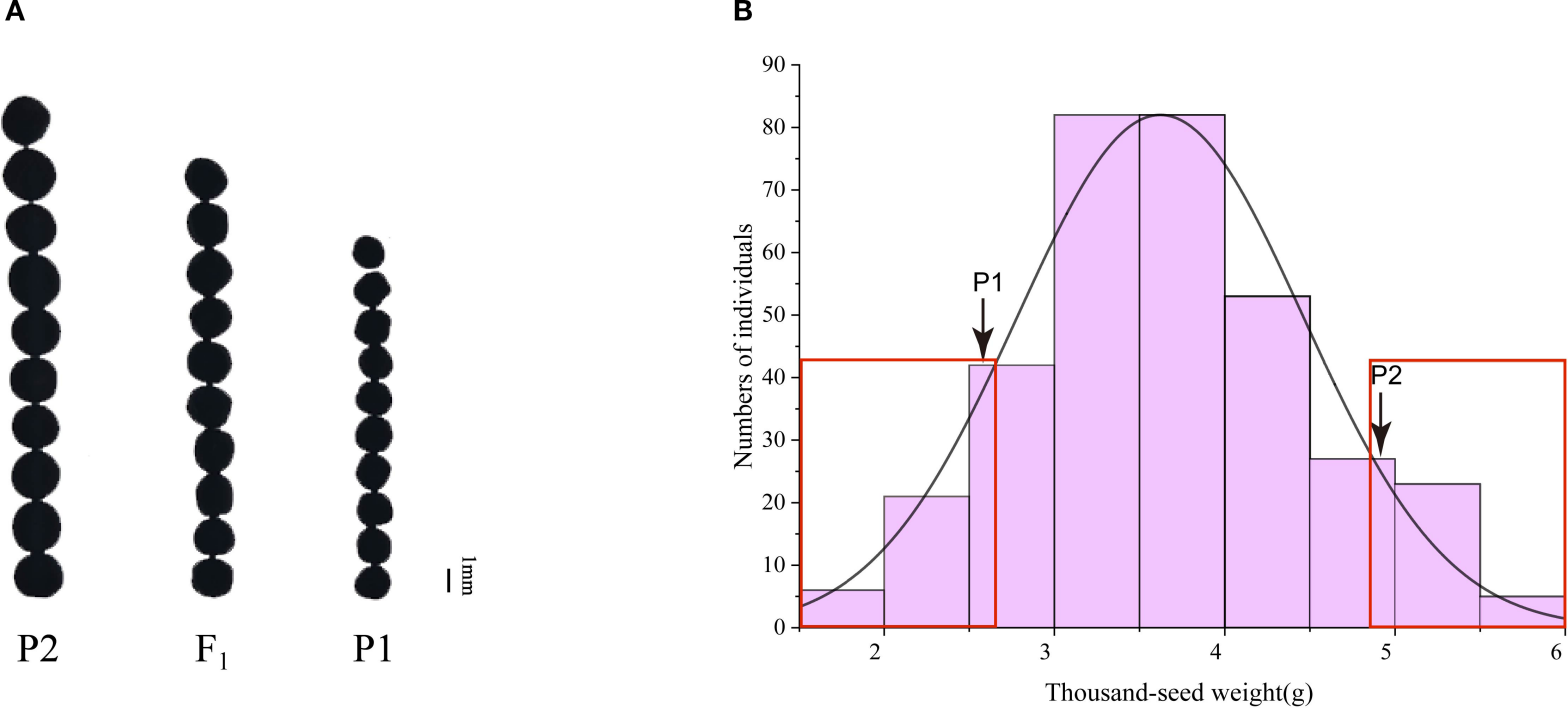
Phenotype of the TSW of two parents, F_1_ plant and F_2_ population. **(A)** Comparisons in seed size in matured seeds of two parents and F_1_ plant. **(B)** Frequency distribution of TSW of 341 individuals in F_2_ population. P1: R140; P2: Zhongshuang 11(ZS11).

To further clarify the genetic pattern of the small seed mutant phenotype, we used the small seed mutant R140 (P1) and ZS11 (P2) as parents to cross and obtain F_1_ progeny, and then F_1_ produced F_2_ population by self-crossing. The seed size of F_1_ plants is intermediate between the two parents (**Figure 3A**), and the TSW of F_2_ population plants was analyzed and exhibited continuous distributions with transgressive segregation, following a normal distribution (**Figure 3B**). These results indicated that the TSW phenotype may be controlled by multiple genes.

### 3.2 BSA-seq analysis for TSW

To identify genomic intervals associated with seed weight phenotype, BSA-seq was performed using 30 extremely large-seed plants and 30 extremely small-seed plants from F_2_ population (**Figure 3B**). After filtering, we obtained 114.15 Gb of clean data, which included 30.06 Gb, 29.76 Gb, 24.35 Gb and 29.98 Gb corresponding to the large seed pool, small seed pool, R140, and ZS11, respectively, yielding average depth of approximately 29×, 28×, 23× and 28×. The sequencing data exhibited excellent quality metrics, with an average Q30 score of 99.16%, an average GC content of 37.19%, and approximately 92.59% of clean reads being properly mapped across all pools (**Supplementary Table 2**). These high-quality datasets provide a reliable foundation for subsequent genetic analyses.

**Figure 4:**
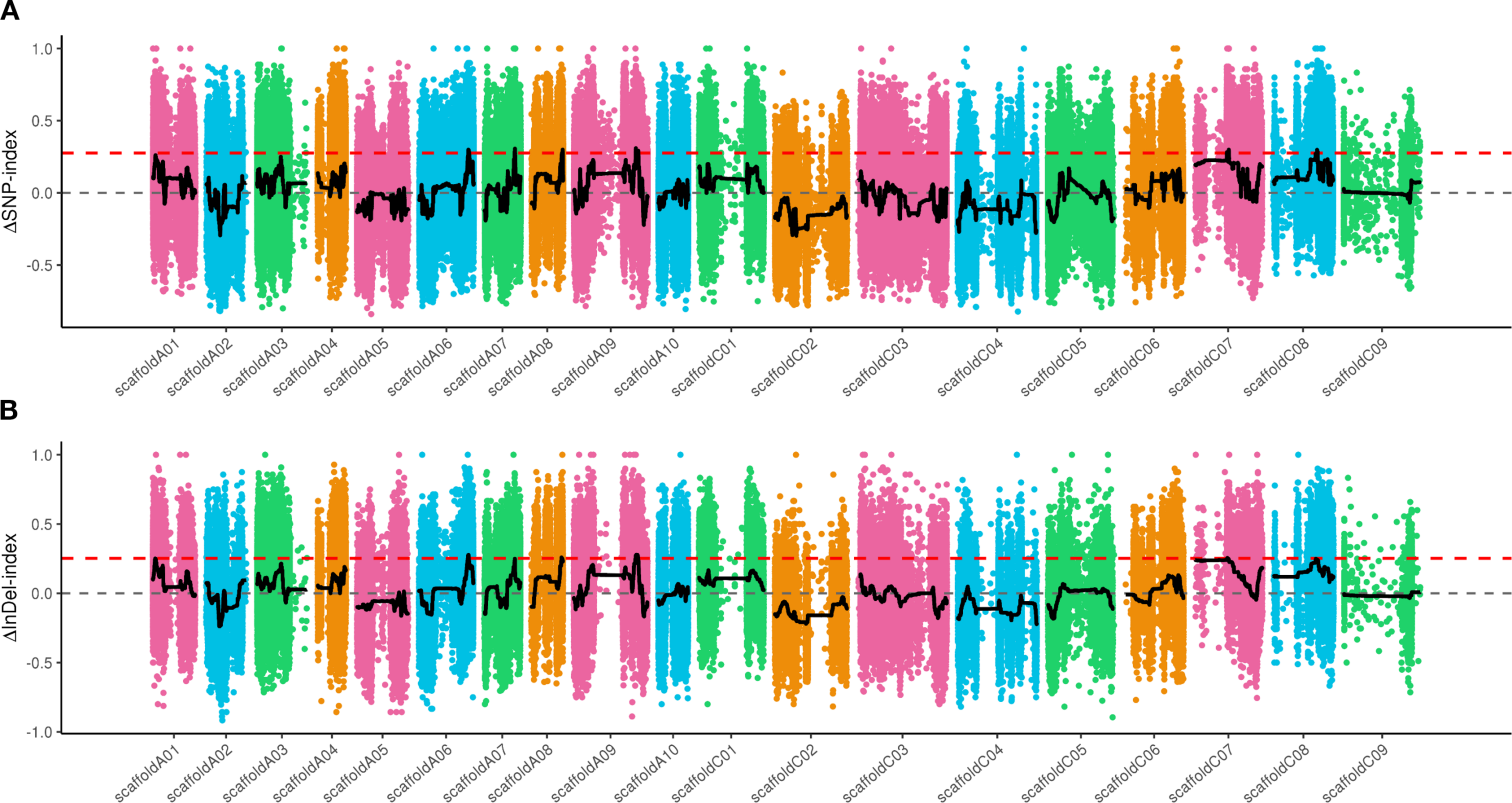
Distribution of SNP and Indel association values on chromosomes. **(A)** the distribution of SNPs. **(B)** the distribution of Indels. The red line represents the 99% CI threshold.

Total of 2,460,870 SNPs and 826,980 Indels were finally obtained by comparison, filtering, and screening of sequencing data among samples. According to Δ (SNP-index), a total of 7 candidate intervals were identified on 6 chromosomes, including A06, A07, A08, A09, C07 and C08 (**Figure 4A**). According to Δ (Indel-index), a total of 4 candidate intervals were identified on 4 chromosomes, including A06, A08, A09 and C07 (**Figure 4B**). By taking the intersection of SNP and Indel associated regions, a total of 5 candidate regions with a physical distance of 1.46Mb were obtained and distributed on Chromosomes A06 (43.88-44.63Mb), A08 (27.63-27.68Mb), A09 (55.32-55.46Mb; 57.33-57.58Mb) and C07 (29.40-29.60Mb). A total of 204 genes were annotated within the candidate regions, including 103 non-synonymous mutant genes and 26 frameshift mutant genes annotated between parents. The annotation results are shown in **Supplementary Table 3**.

### 3.3 RNA-seq data analysis

To elucidate the molecular mechanisms underlying the phenotypic differences between large and small seeds in *Brassica napus*. Six cDNA libraries with three biological replicates were sequenced. After filtering out all low-quality sequences and adapters, 5.373Gb, 5.383Gb, 5.382Gb, 5.371Gb, 5.431Gb and 5.423Gb clean data was obtained, respectively. Q30 ratio was above 96.30% (**Supplementary Table 4**). More than 84.49% of clean reads were successfully mapped to the reference genome for further analysis (**Supplementary Table 5**).

**Figure 5:**
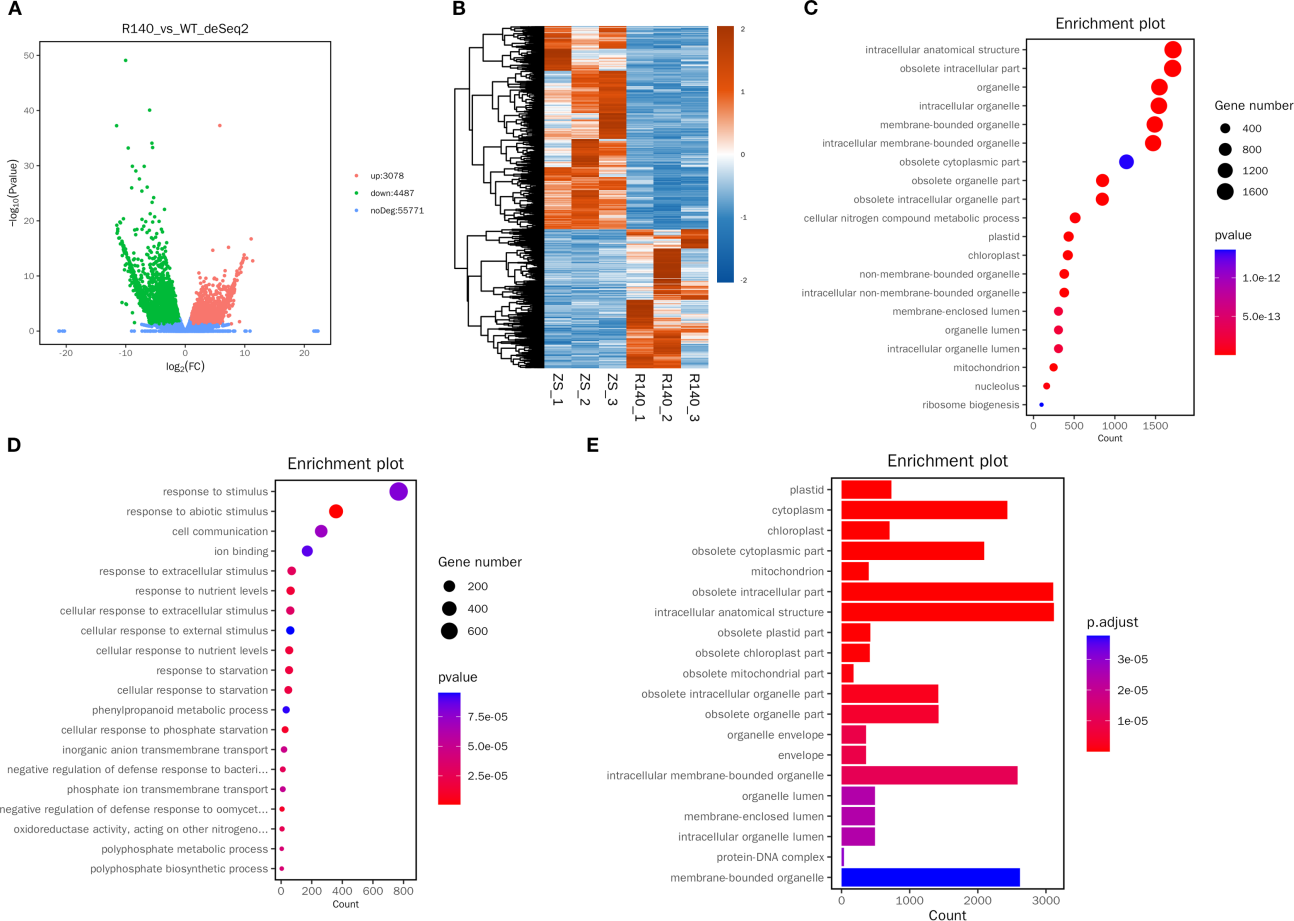
Analysis of DEGs identified among R140 and Zhongshuang 11(ZS11) seeds. **(A)** Volcano diagram showing the number of no differentially expressed genes and DEGs between two parents. **(B)** Heatmap showing the DEGs between two parents. **(C)** Top 20 GO functional enrichment of the up-regulated DEGs. **(D)** Top 20 GO functional enrichment of the down-regulated DEGs. **(E)** KEGG pathway enrichment analysis of DEGs.

There were totally 70,399 expressed genes were identified separately in the developing seeds between ZS11 and R140. A total of 7,565 DEGs were identified between the small-seed and large-seed plants, of which 3,078 were up-regulated and 4,487 down-regulated (**Figures 5A, B**). qRT-PCR analysis was conducted on 7 randomly selected DEGs, and the expression differences could be confirmed for most of these genes (**Supplementary Figure 2**), indicating that the RNA-seq data was accurate and reliable.

GO enrichment analysis showed that up-regulated DEGs were mainly enriched in the main pathways of intracellular anatomical structure and obsolete intracellular (**Figure 5C**), and down-regulated DEGs are mainly enriched in the pathway of response to stimulus (**Figure 5D**).

Meanwhile, KEGG pathway enrichment analysis showed that 7,565 DEGs participated in 126 pathways (**Supplementary Table 6**). Of these, 11 pathways were significantly enriched including glutathione metabolism, arginine and proline metabolism, pyrimidine metabolism, ribosome biogenesis in eukaryotes, purine metabolism, pyruvate metabolism, ribosome, sulfur relay system, glycerolipid metabolism, photosynthesis and monoterpenoid biosynthesis (**Figure 5E**).

### 3.4 Association analysis between BSA-seq and RNA-seq data

**Figure 6:**
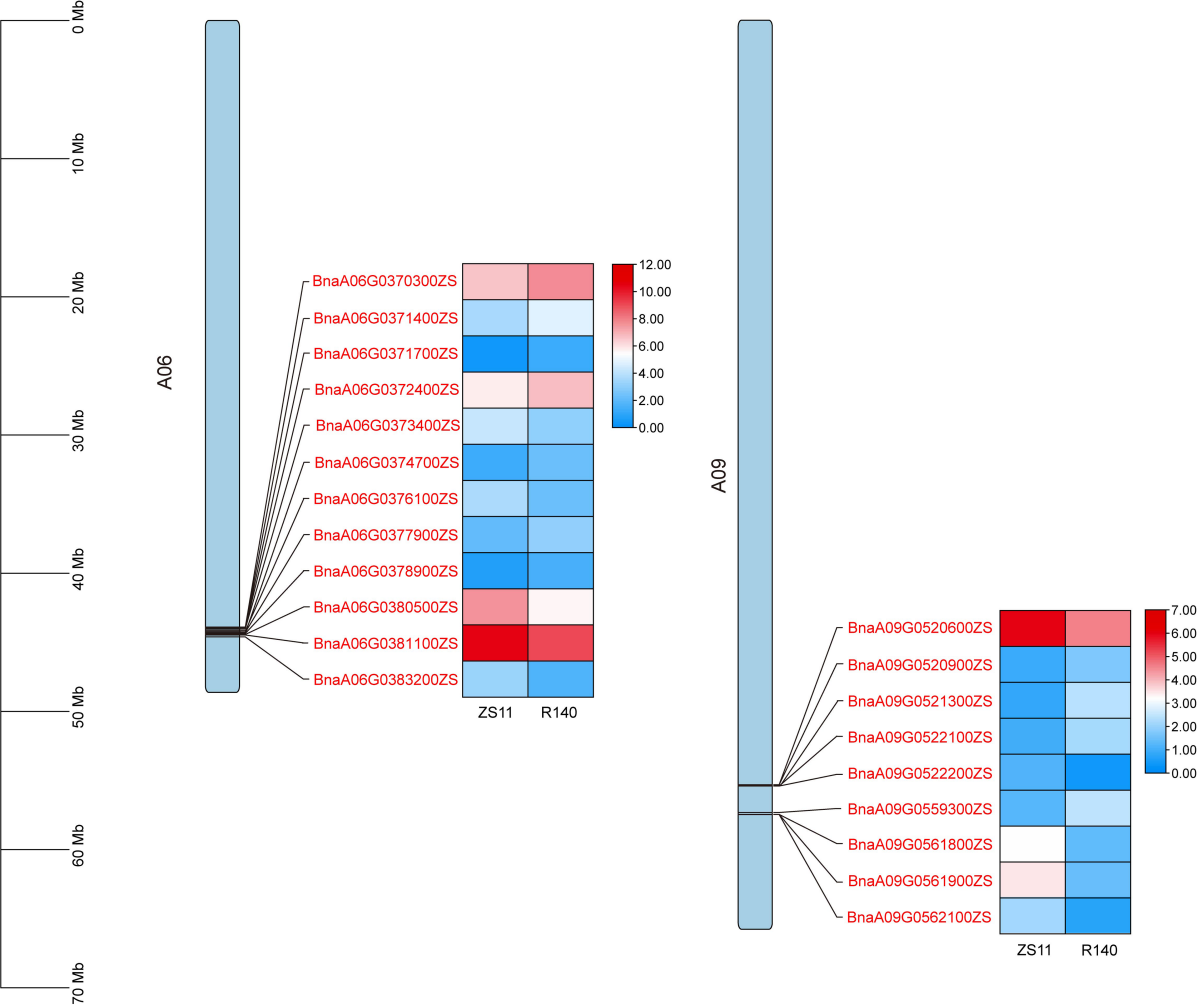
The expression patterns of the 21 candidate genes screened from 5 candidate intervals related to seed weight phenotype based on RNA-seq. The position distribution map of genes on chromosomes and the heat map of expression were drawn using TBtools (Chen et al., 2023).

**Figure 7:**
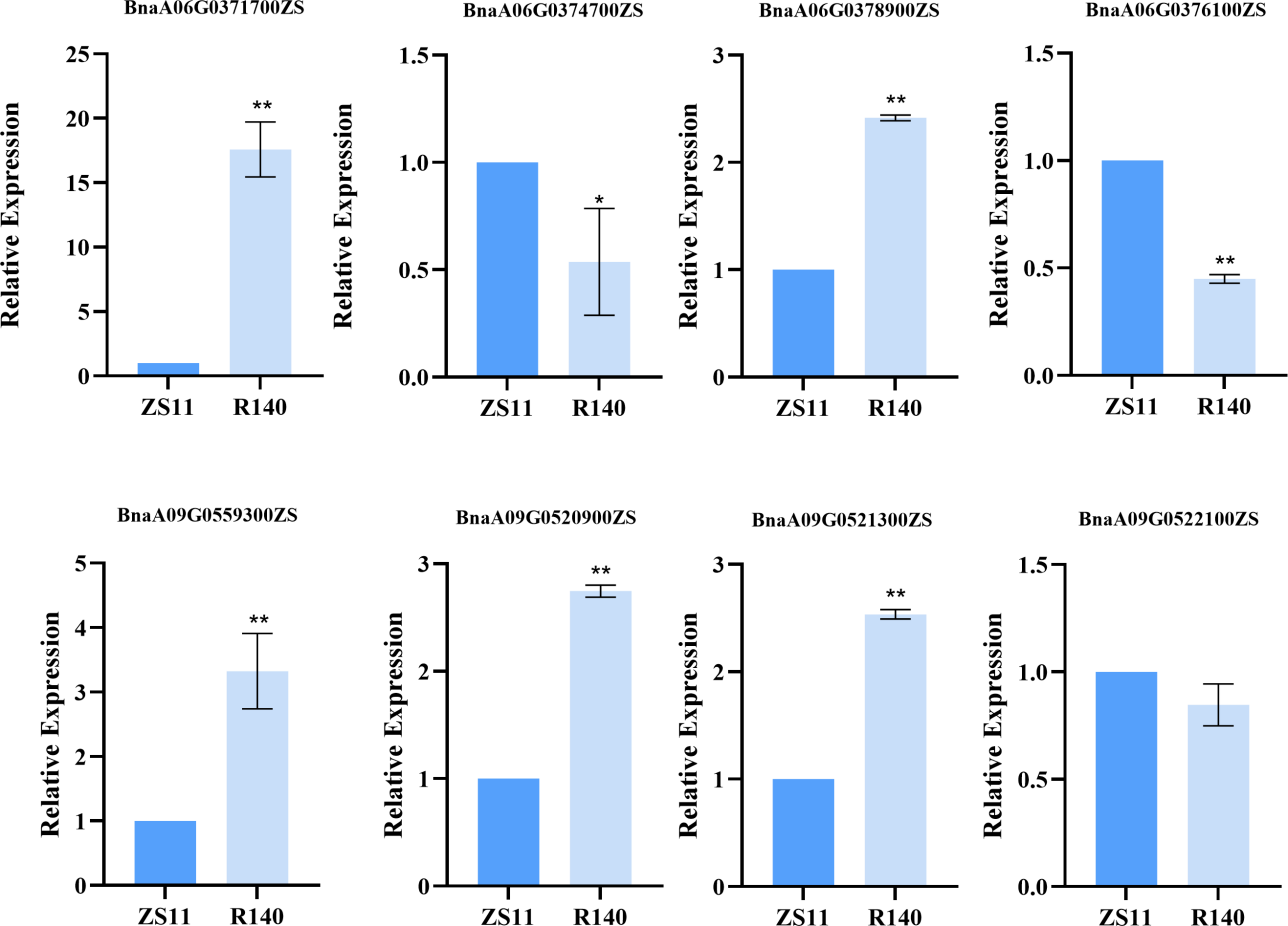
Relative expression of candidate genes in the R140 and Zhongshuang 11 (ZS11) as detected by qRT-PCR.

To rapidly identify candidate genes associated with the R140 seed weight phenotype, we conducted a comprehensive association analysis that integrated BSA-seq and RNA-seq results. Based on RNA-seq data, a total of 21 DEGs were identified from 5 candidate intervals related to seed weight (**Figure 6**, **Table 1**). By aligning BSA-seq reads from R140 and ZS11 to the *B. napus* reference genome, we further characterized these DEGs, identifying 3 genes (*BnaA06G0376100ZS*, *BnaA09G0520900ZS* and *BnaA09G0521300ZS*) with frameshift mutations, 4 genes (*BnaA06G0371700ZS*, *BnaA06G0374700ZS*, *BnaA06G0378900ZS* and *BnaA09G0522100ZS*) with nonsynonymous mutations, and 1 gene (*BnaA09G0559300ZS*) exhibiting an upstream mutation. Therefore, by combining the results of BSA-seq with RNA-seq, these 8 genes may be the candidate genes within the targeted region. To verify the expression of the candidate genes in R140 and ZS11 samples, we performed qRT-PCR on these 8 genes. The expression profiles in qRT-PCR were consistent with those in RNA-seq, except for *BnaA09G0522100ZS*. It was worth noting that *BnaA09G0559300ZS*, which encodes an auxin response factor 18-like (*BnARF18*), was found that there was a very significant difference in expression levels between two parents. Using ZS11 as a control, the expression level of *BnARF18* in R140 was significantly increased, indicating negative regulation of seed weight (**Figure 7**). This result was consistent with the findings reported by Liu et al. (2015). Four genes have similar expression trends with *BnARF18*, including *BnaA06G0371700ZS*, *BnaA06G0378900ZS*, *BnaA09G0520900ZS* and *BnaA09G0521300ZS*. Based on the annotation information, *BnaA06G0371700ZS* encodes hypothetical protein BRARA, *BnaA06G0378900ZS* encodes bidirectional sugar transporter SWEET4, *BnaA09G0520900ZS* encodes ribosomal lysine N-methyltransferase 3, and *BnaA09G0521300ZS* encodes WAT1 related protein, all of which were not found to be enriched in pathways related to seed size or seed weight.

**Table 1:** Annotation information of 20 DEGs screened from 3 BSA-seq candidate intervals.

ID	DEG Type	Nr
*BnaA06G0370300ZS*	Up	XP_009151764.1 protein RALF-like 27 [*Brassica rapa*]
*BnaA06G0371400ZS*	Up	XP_009151777.1 histidine-containing phosphotransfer protein 2 [*Brassica rapa*]
*BnaA06G0371700ZS*	Up	RID59895.1 hypothetical protein BRARA_F03087 [*Brassica rapa*]
*BnaA06G0372400ZS*	Up	XP_013644493.1 uncharacterized protein LOC106349125 [*Brassica napus*]
*BnaA06G0373400ZS*	Down	XP_013644506.1 pectinesterase 31 [*Brassica napus*]
*BnaA06G0374700ZS*	Up	XP_013644521.1 transcription factor MYB30 [*Brassica napus*]
*BnaA06G0376100ZS*	Down	KAG5394773.1 hypothetical protein IGI04_024736 [*Brassica rapa*]
*BnaA06G0377900ZS*	Up	VDC68439.1 unnamed protein product, partial [*Brassica rapa*]
*BnaA06G0378900ZS*	Up	XP_009151901.1 bidirectional sugar transporter SWEET4 [*Brassica rapa*]
*BnaA06G0380500ZS*	Down	XP_013644446.1 50S ribosomal protein L12-1, chloroplastic [*Brassica napus*]
*BnaA06G0381100ZS*	Down	XP_013644620.1 chlorophyll a-b binding protein 2.4, chloroplastic-like [*Brassica napus*]
*BnaA06G0383200ZS*	Down	XP_022543597.1 protein WVD2-like 7 [*Brassica napus*]
*BnaA09G0520600ZS*	Down	XP_013663422.1 14 kDa zinc-binding protein-like [*Brassica napus*]
*BnaA09G0520900ZS*	Up	XP_013663409.1 ribosomal lysine N-methyltransferase 3-like [*Brassica napus*]
*BnaA09G0521300ZS*	Up	XP_013663405.1 WAT1-related protein At3g56620 [*Brassica napus*]
*BnaA09G0522100ZS*	Up	XP_013663391.1 rhomboid-like protein 20 isoform X1 [*Brassica napus*]
*BnaA09G0522200ZS*	Down	XP_013663390.1 uncharacterized protein LOC106368059 [*Brassica napus*]
*BnaA09G0559300ZS*	Up	Auxin response factor 18 [*Brassica napus*]
*BnaA09G0561800ZS*	Down	XP_009116880.1 pectin acetylesterase 6 [*Brassica rapa*]
*BnaA09G0561900ZS*	Down	XP_013663930.1 pectin acetylesterase 6 [*Brassica napus*]
*BnaA09G0562100ZS*	Down	NP_001303222.1 charged multivesicular body protein 7-like [*Brassica napus*]

### 3.5 Natural variation of *BnARF18* promoter sequence led to differences in expression levels

**Figure 8:**
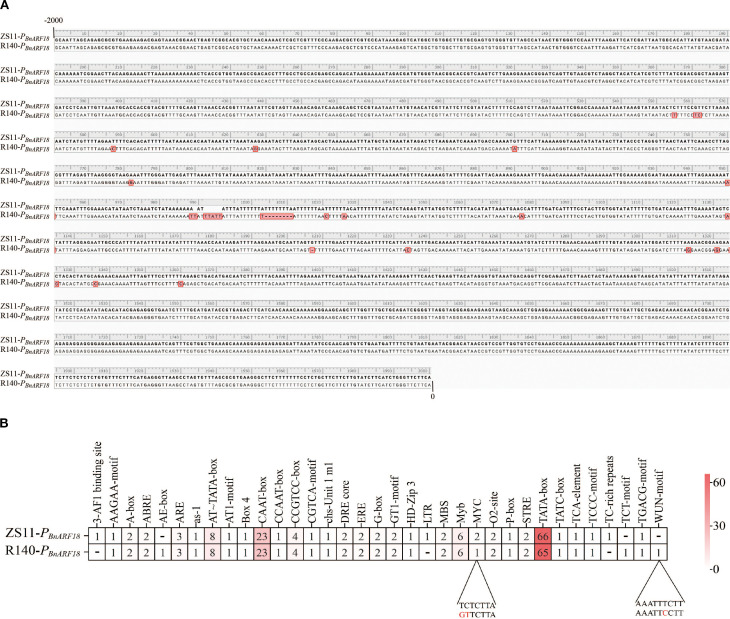
Comparison of promoter sequences and promoter elements of *BnARF18* gene between R140 and Zhongshuang 11 (ZS11). **(A)** promoter sequence. **(B)** promoter elements with variations.

To further verify the reasons for the differential expression levels of *BnARF18* gene between parents, the promoter sequence, full-length sequence, and protein sequence of the *BnARF18* gene from two parents were cloned. Comparison of the parental *BnARF18* promoters, proteins and full-length gene sequence showed that there were in total 21 SNPs and Indels in *ARF18* promoter between ZS11 and R140 (**Figure 8A**), however, there was no difference in protein sequence and full-length sequence. It indicated that the differential expression level of the *BnARF18* gene was caused by variations in the promoter sequence between two parents. By analyzing the functions of the promoter elements with variations, it was found that the MYC-motif, which was involved in the regulation of gene expression, and the WUN-motif, which regulated cell differentiation and proliferation, might be the key motifs that affected the difference in expression levels of *BnARF18* between two parents (**Figure 8B**).

## 4 Discussion

Seed weight is one of the three major factors that contribute to the yield per plant (silique number per plant, seed number per silique and seed weight) in *Brassica napus*, and an important breeding objective. So far, although many QTLs related to seed weight have been obtained in *Brassica napus*, the cloning and functional research of seed weight related genes have not made breakthrough progress due to the complexity of the *Brassica napus* genome (AACC, allotetraploid) (Fan et al., 2010; Cai et al., 2014). To date, only a few genes, including *ARF18* and *BnaA9.CYP78A9*, have been identified via map-based cloning (Liu et al., 2015; Shi et al., 2019). Several others, such as *BnDA1*, *BnaC3.UPL3*, *BnRRF*, *BnEOD1*, *BnaWRKY10*, *BnUBP15*, and *BnaARF2*, have been characterized through homology-based cloning (Wang et al., 2017; Miller et al., 2019; Hu et al., 2022; Gu et al., 2023a, b; Tian et al., 2023). Consequently, most genetic studies on seed weight in *B. napus* remain at the QTL level, with multiple mapping populations, including DH, F_2_, and RILs, consistently revealing loci for thousand-seed weight (TSW) (Fan et al., 2010; Zhang et al., 2011; Yang et al., 2012; Wang et al., 2020b; Meng et al., 2025). Genome-wide association studies (GWAS) have further identified numerous significant SNPs associated with TSW across various chromosomes (Cai et al., 2014; Li et al., 2014; Dong et al., 2018), underscoring the polygenic nature and extensive genetic variation underlying this trait. To our knowledge, some mutants that affect seed weight have been found in *B. napus*, such as zy72360 and R1 (Liu et al., 2015), G-42 and 7-9 (Geng et al., 2018), etc. However, in this study, R140 not only exhibited smaller seed mutations compared to ZS11, but also showed lower plant height, shorter silique and lower yield per plants. Besides, the decrease in yield per plant was mainly due to the reduction in seed weight, rather than the number of siliques per plant or the number of seeds per silique. Therefore, R140 is an excellent resource for understanding the molecular mechanism of seed weight and studying how to affect the yield of Brassica napus by regulating seed weight.

In this study, an integrated approach combining Bulked Segregant Analysis sequencing (BSA-seq) and RNA sequencing (RNA-seq) was applied to efficiently identify candidate genes regulating seed weight. In total, we screened 5 candidate genomic regions including 204 gene associated with seed weight and integrated transcriptomic data to pinpoint 21 differentially expressed genes (DEGs) within these regions. These DEGs represent high-confidence candidates for further functional validation and highlight the power of multi-omics integration in advancing the genetic dissection of complex traits in polyploid crops. The discovery of 5 seed weight related intervals provides useful molecular markers for subsequent seed weight molecular assisted breeding in *Brassica napus*.

Seed weight is a critical factor affecting the yield of *Brassica napus*. Mapping the genes related to seed weight on chromosomes can contribute to uncovering the genetic mechanism of yield and provide an important theoretical basis for high-yield *Brassica napus* breeding. Many studies have focused on this area, and different research findings show diverse results in the chromosomal intervals of related QTL mapping. In this study, the candidate regions for seed weight screened by BSA-seq are located on chromosomes A06, A08, A09, and C07. Among them, chromosome A09 has always been considered the chromosome where the major genes for seed weight are mainly distributed. Comparing with the results of previous studies, the first successfully cloned gene related to seed weight is *ARF18* in *Brassica napus*, which was located on chromosome A09. The location of this gene coincided with the location of the major candidate loci of A09 in this study, and *ARF18* gene was also found in the candidate region of chromosome A09 in this study (Liu et al., 2015), which was different from the regions for the thousand-seed weight trait mapped on chromosome A09 in other studies (Yang et al., 2012; Geng et al., 2016; Dong et al., 2018; Song et al., 2020). The candidate region of chromosome A07 in this study was inconsistent with the previously reported candidate region for seed weight on chromosome A07 (Raboanatahiry et al., 2018). Compared with previous research results, candidate regions on A06 and A08 in this study were two new candidate loci for seed weight. These discoveries not only expand the distribution map of *Brassica napus* seed weight genes on chromosomes, but also provide important theoretical bases and gene resources for in-depth analysis of the genetic mechanism of seed weight and the implementation of molecular marker-assisted breeding.

Through analysis of differentially expressed genes in the transcriptomic data and functional annotation information of genes within candidate regions, it was found that a gene belonging to the RALFs (Rapid alkalinization factors) family was identified. This multifunctional plant cytokine can rapidly induce extracellular pH elevation in plant cells, is recognized by the receptor-like kinase FER (FERONIA), and participates in various biological processes including plant growth and development, cell elongation, stress responses, and immune reactions (He et al., 2023). A reported seed weight gene *ARF18* was also screened in the candidate regions by BSA-seq and RNA-seq in this study. qRT-PCR data indicated the expression level of *BnARF18* in R140 was significantly increased, indicating negative regulation of seed weight, which was consistent with the previous findings by Liu et al. (2015). We compared the sequences of *BnARF18* promoter, proteins and full-length gene sequence and the results showed that there were 21 SNPs and Indels in promoter, and no difference in protein sequence and full-length sequence between ZS11 and R140 (**Figure 8**). It indicated that the differential expression of *BnARF18* was likely due to variations in the promoter, which was different from the previously reported by Liu et al. (2015) and also has not been reported in other studies before. The *ARF18* gene (a seed weight gene previously validated) in small seed mutant line R1 was observed to harbor a 165-bp core fragment in its exon, which played a crucial role in homodimers formation for silique elongation in *Brassica napus* (Liu et al., 2015). Above all, it implied that R140 and R1 might be different variation types of the same gene associated with seed weight in *Brassica napus*.

Functional analysis of the polymorphic promoter elements suggested that the MYC-motif, involved in gene expression regulation, and the WUN-motif, associated with cell differentiation and proliferation (Li et al., 2024b; Siebertz et al., 1989), might be the key motifs responsible for the expression divergence between the two parents in this study. However, further study will focus on and explore which element is the functional element by truncating the *BnARF18* promoter, analyzing promoter activity, and verifying the proteins that interact with its promoter and help clarify the molecular mechanisms and regulatory pathways affects seed weight by *BnARF18* in the future.

## Data Availability

The datasets of BSA-seq and RNA-seq data generated during the current study are available in NCBI, Accession number: PRJNA1299313 and PRJNA1295376.
