# Supplementary material for: Combined BSA-seq and RNA-seq approaches reveal candidate genes associated with seed weight in Brassica napus
Source: Front Plant Sci. 2025 Sep 16;16:1678464. doi: 10.3389/fpls.2025.1678464 (PMC12481171; doi:10.3389/fpls.2025.1678464)
Supplement: Supplementary file 1 [file DataSheet1.pdf]

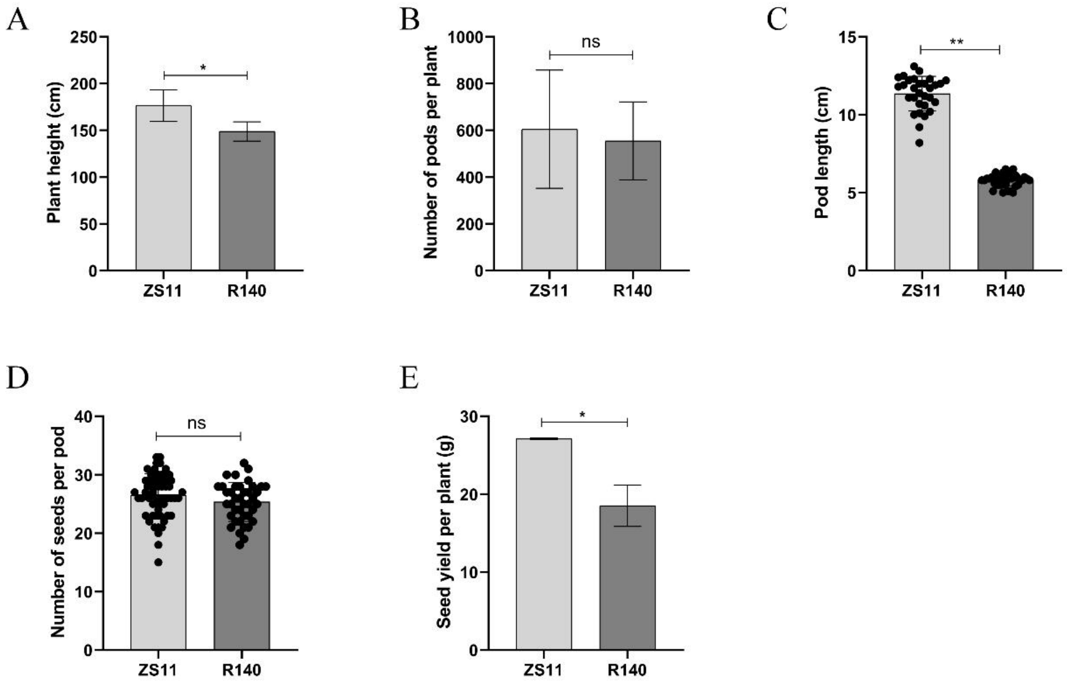

Supplementary Figure 1. Statistics and comparison of five yield related traits between two parents, ZS11 and *R140*. (A) plant height (n=5). (B) number of pods per plant (n≥20). (C) pod length (n≥20). (D) number of seeds per pod (n≥20). (E) seed yield per plant (n=5). Values are means ± SD. Student's t-test was used to generate the p-values. ** means p <0.01,* means p <0.05, ns means no significance.
