# Supplementary material for: Combined BSA-seq and RNA-seq approaches reveal candidate genes associated with seed weight in Brassica napus
Source: Front Plant Sci. 2025 Sep 16;16:1678464. doi: 10.3389/fpls.2025.1678464 (PMC12481171; doi:10.3389/fpls.2025.1678464)
Supplement: Supplementary file 2 [file DataSheet2.pdf]

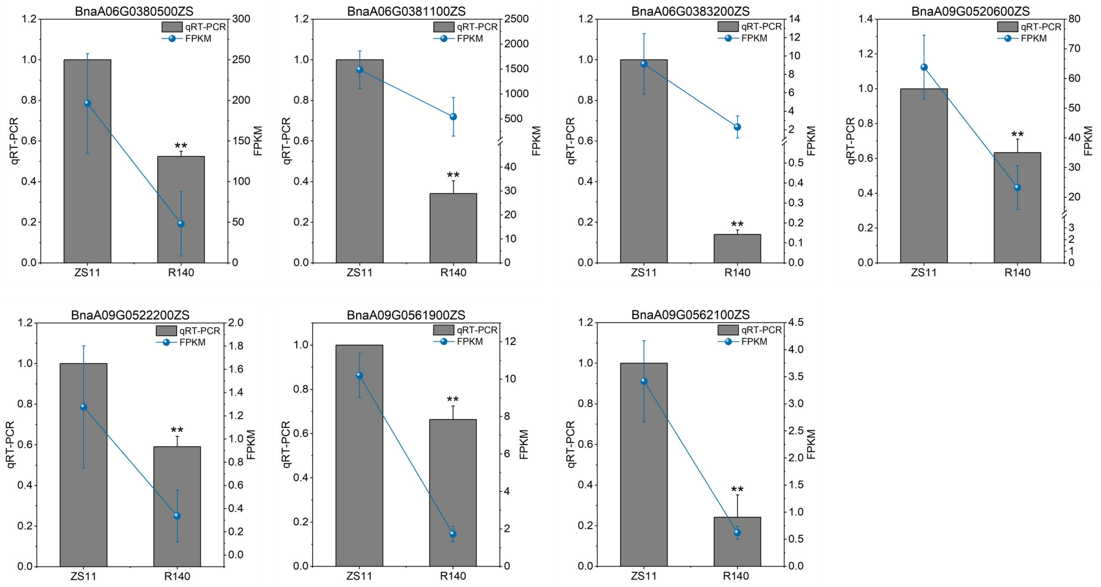

Supplementary Figure 2. The expression changes of randomly selected seven genes confirmed by qRT-qPCR analysis to verify transcriptome data between ZS11 and R140. The comparative log_2FPKM and $2^{-\Delta\Delta Ct}$ values of ZS11 were used as the control for normalization. Means ± SDs, n = 3 biological replicates.
